# Therapeutic Options of Crystallin Mu and Protein Disulfide Isomerase A3 for Cuprizone-Induced Demyelination in Mouse Hippocampus

**DOI:** 10.1007/s11064-024-04227-4

**Published:** 2024-08-20

**Authors:** Kyu Ri Hahn, Hyun Jung Kwon, Dae Won Kim, In Koo Hwang, Yeo Sung Yoon

**Affiliations:** 1 Department of Anatomy and Cell Biology, College of Veterinary Medicine, and Research Institute for Veterinary Science, Seoul National University, Seoul, 08826 South Korea; 2 Department of Biochemistry and Molecular Biology, Research Institute of Oral Sciences, College of Dentistry, Gangneung-Wonju National University, Gangneung, 25457 South Korea; 3 Department of Biomedical Sciences, and Research Institute for Bioscience and Biotechnology, Hallym University, Chuncheon, 24252 South Korea

**Keywords:** Cuprizone, Demyelination, Proteomics, CRYM, PDIA3, Hippocampus, Neurogenesis

## Abstract

This study investigates the changes in hippocampal proteomic profiles during demyelination and remyelination using the cuprizone model. Employing two-dimensional gel electrophoresis and liquid chromatography-tandem mass spectrometry for protein profiling, we observed significant alterations in the expression of ketimine reductase mu-crystallin (CRYM) and protein disulfide isomerase A3 precursor (PDIA3) following exposure to and subsequent withdrawal from cuprizone. Immunohistochemical staining validated these protein expression patterns in the hippocampus, revealing that both PDIA3 and CRYM were downregulated in the hippocampal CA1 region during demyelination and upregulated during remyelination. Additionally, we explored the potential protective effects of CRYM and PDIA3 against cuprizone-induced demyelination by synthesizing cell-permeable Tat peptide-fusion proteins (Tat-CRYM and Tat-PDIA3) to facilitate their crossing through the blood–brain barrier. Our results indicated that administering Tat-CRYM and Tat-PDIA3 mitigated the reduction in proliferating cell and differentiated neuroblast counts compared to the group receiving cuprizone alone. Notably, Tat-PDIA3 demonstrated significant effects in enhancing myelin basic protein expression alongside phosphorylation of CREB in the hippocampus, suggesting its potential therapeutic role in the prevention or treatment of demyelination, and by extension, in conditions such as multiple sclerosis.

## Introduction

The hippocampus—one of the important parts of the limbic system—plays pivotal roles in learning and memory. In particular, it performs crucial functions in transforming short-term memory into long-term memory and, likewise, in spatial cognition [1, 2]. Adult hippocampal neurogenesis (AHN) involves the continuous production of neural stem cells in specific brain regions such as the subventricular zone of the lateral ventricle and subgranular zone (SGZ) of the dentate gyrus (DG) within the hippocampus [3, 4]. The DG consists of three layers: molecular layer (ML), granular cell layer (GCL), and polymorphic layer (PoL). The ML contains dendrites of granule cells and axons derived from the entorhinal cortex. The GCL is formed by the cell bodies of granule cells, where dendrites enter the ML and accept input signals from the perforant pathway of the entorhinal cortex. The PoL, the deepest layer of the DG, constitutes the axons of granule cells and mossy fibers with abundant intervening neurons [5, 6]. The CA1 subregion's association with adult neurogenesis adds a significant dimension to the understanding of the hippocampus, a vital brain structure responsible for memory formation, spatial navigation, and other cognitive functions, offering potential insights into the mechanisms underlying learning and memory processes [7].

Newly generated granule cells are produced from neural stem cells in the SGZ through the processes of proliferation, differentiation, and maturation. Type 1 cells are called radial glial cells because of their morphology. Type 2 cells are characterized by the most potent proliferative potential and are believed to be immediate precursor cells during transition from glial to neuronal lineages. Finally, type 3 cells (i.e., neuroblasts) depart the cell cycle and differentiate into immature neurons. Thereafter, the maturation of newly generated neurons is characterized by rapid axonal and dendritic growth and elevated synaptic plasticity. Axons of mature neurons expand into the pyramidal cells of hippocampal CA3 region [8, 9], and these integrated neurons are involved in the function of neural circuitry in the hippocampus [10]. Myelin allows neuron to transmit information faster, because the myelination of axons propagates the action potential by jumping node to node.

Multiple sclerosis (MS) is characterized by autoimmune-mediated attack on the myelin sheath, which hinders the ability of nerves to transmit messages properly, with the risk of subsequent degeneration. In many patients with MS, nerve damage and extensive demyelination in the hippocampus have been reported as pathological features [11]. Although drugs may help reduce the disease progression, the symptoms of MS cannot be reversed, and the mechanisms underlying tissue damage and the ameliorative effects of these drugs are only partly understood.

Cuprizone (CPZ), a copper chelator, has been widely used to induce the damage and loss of myelin and oligodendrocytes in the brain [12], including the hippocampus [13]. Repeated exposure to CPZ is indicative of gray matter demyelinating disease, resembling the condition in MS, which causes amnesia [14, 15]; it has also been used in the process of remyelination. Meanwhile, termination of CPZ has been reported to result in strong remyelination within a few days [16]. CPZ-induced mouse models resulted in a significant decrease in hippocampal MBP levels, which was associated with reduced cell proliferation and neuroblast differentiation in the DG [13, 17]. Several approaches exist for the analysis of CPZ-induced demyelination in multiple brain regions [18, 19]. For instance, Szilagyi et al. (2020) used proteomics to study demyelination and remyelination in the corpus callosum. However, no systemic approaches have been employed to assess hippocampal protein changes in the presence of clear CPZ-induced demyelination [12] and AHN reduction [20].

In this study, we hypothesized that exposure to CPZ causes specific modulations in the protein profiles of the hippocampus, which are involved in the processes of demyelination and subsequent remyelination. To investigate this, two-dimensional gel electrophoresis (2DE) and liquid chromatography-tandem mass spectrometry (LC–MS/MS)-based quantification methods were applied to evaluate evident protein changes following the intake of a CPZ-supplemented and/or normal diet. We specifically sought to examine the modulations in hippocampal protein profiles during demyelination and remyelination induced by CPZ exposure for 8 weeks or CPZ exposure for 5 weeks, followed by CPZ termination for 3 weeks. Among the identified and significantly altered proteins, those whose expression was suppressed after CPZ intake but promoted after CPZ termination were further analyzed. This analysis was crucial for assessing their effects on CPZ-induced demyelination and considering the implications for the treatment of demyelination diseases.

## Materials and Methods

### Experimental Animals

Four-week-old male C57BL/6N mice were obtained from the Central Lab Animal Inc. (Seoul, South Korea). All animals were maintained at 22 ± 1℃ and 60% ± 5% humidity under a 12 h/12 h light/dark cycle; food and water were provided ad libitum until the end of experiment. All experimental protocols were approved by the Institutional Animal Care and Use Committee of the Seoul National University (SNU-190314–12-2, SNU-190906–2, and SNU-210917–2). Animal handling and management conformed to guidelines established under the current international laws and policies (NIH Guide for the Care and Use of Laboratory Animals, NIH Publication No. 85–23, 1985, revised 1996). All experimental processes were performed to minimize the number of animals used and the suffering of experimental animals caused by the research procedure. For 2DE, LC–MS/MS, and validation of proteins, the animals were divided into three groups (*n* = 20 per group for 2DE and LC–MS/MS as well as *n* = 5 per group for protein validation): a group receiving normal chow diet for 8 weeks (CTL), a group receiving CPZ-supplemented diet for 8 weeks (Cu), and a group receiving normal diet for 3 weeks following CPZ-supplemented diet for 5 weeks (Cu + N). The CPZ-supplemented diet contained 0.2% CPZ (Sigma-Aldrich, St. Louis, MO, USA) in AIN-76-based chow diet, as described in a previous study [17].

To investigated the protective effects of ketimine reductase mu-crystallin (CRYM) and protein disulfide isomerase A3 precursor (PDIA3) against cuprizone-induced demyelination, the animals were divided into four groups (*n* = 10 per group): CTL, Cu, CPZ-supplemented diet-fed mice with Tat-CRYM injection (Cu + CRYM), and CPZ-supplemented diet-fed mice with Tat-PDIA3 injection (Cu + PDIA3). The CPZ-supplemented diet was provided for 8 weeks, and Tat-CRYM (3 mg·kg^−1^) or Tat-PDIA3 (3 mg·kg^−1^) was administered intraperitoneally once a day for the 4 final weeks because our unpublished and published studies showed significant intracellular delivery of these proteins into hippocampus or hippocampal DG of normal and ischemic animals [21]. Tat-CRYM and Tat-PDIA3 was dissolved in 10% glycerol and CTL and Cu group was received same volume of 10% glycerol once a day for the 4 final weeks.

### Two-Dimensional gel Electrophoresis

Following treatment with CPZ diet for 8 weeks or CPZ diet for 5 weeks, followed by normal diet for 3 weeks, the mice were anesthetized with a mixture of alfaxalone (75 mg/kg; Careside, Seongnam, South Korea) and xylazine (10 mg/kg; Bayer Korea, Seoul, South Korea). Hippocampal tissues were isolated from the brains and suspended in a mixture of buffer described elsewhere [22]. 2DE was performed as follows [23]. First, sample aliquots in buffer (7 M urea, 2 M thiourea, 4.5% 3-((3-cholamidopropyl) dimethylammonio)-1-propanesulfonate, 100 mM dithioerythritol, and 40 mM Tris [pH 8.8]) were applied to immobilized pH 3–10 nonlinear gradient strips (Amersham Biosciences, Uppsala, Sweden). Isoelectric focusing was regulated at 80,000 Vh. For second dimension, electrophoresis was performed with 9–16% linear gradient polyacrylamide gels (18 cm × 20 cm × 1.5 mm) at constant 40 mA per gel for approximately 5 h. For protein fixation, the gels were incubated in 40% methanol and 5% phosphoric acid for 1 h and stained using Coomassie brilliant blue GG-250 for 12 h. After de-staining with distilled water (DW), the gels were scanned in a densitometer (GS710; Bio-Rad, Richmond, CA, USA) and converted into electronic files. The results were analyzed with Image Master Platinum 5.0 (Amersham Biosciences).

### Peptide Analysis Using LC–MS/MS

For peptide analysis, Easy n-LC (Thermo Fisher, San Jose, CA, USA) and LTQ Orbitrap XL mass spectrometer (Thermo Fisher) equipped up with a nano-electrospray source were used. Using a C18 nanopore column (150 mm × 0.1 mm, 3 μm pore size; Agilent), the samples were separated. For LC separation, mobile phase A (0.1% formic acid and 3% acetonitrile in deionized water) and mobile phase B (0.1% formic acid in acetonitrile) were used. The chromatography gradient was scheduled for a linear increase from 0% B to 60% B in 9 min, 60% B to 90% B in 1 min, and 3% B in 5 min at the flow rate of 1,800 nL·min-1. The mass spectra were obtained through data-dependent acquisition with a full mass scan (380–1700 m/z) and 10 MS/MS scans. For MS1 full scans, the orbitrap resolution was 15,000 and the automatic gain control (AGC) was 2 × 10^5^; for MS/MS with LTQ, AGC was 1 × 10^4^.

### Database Search

Using the MASCOT search engine (Matrix Science Inc., Boston, MA, USA), peptide sequences present in the protein sequence database were identified. The database search criteria were as follows: taxonomy = *Mus musculus*; fixed modification = carbamidomethylation at cysteine residues; variable modification = oxidization at methionine residues; maximum allowed missed cleavage = 2; MS tolerance = 10 ppm; and MS/MS tolerance = 0.8 Da. The peptides were processed at a significance threshold of p < 0.05.

The “score” in each table is the MASCOT score, calculated as follows: SCORE = −10 × ln10(P), where P is the molecular weight search score.

### Expression Vector Construction

To facilitate the intracellular delivery and blood–brain barrier crossing of the CRYM and PDIA3 proteins in the mouse hippocampus, Tat-CRYM and Tat-PDIA3 were prepared as it proved in the previous paper [24, 25]. As described previously [24, 25], polymerase chain reaction (PCR) was performed using primers for human CRYM and PDIA3 cDNAs. The PCR product was subcloned into a TA cloning vector and ligated to a Tat expression vector. Each of the plasmids with Tat-CRYM and Tat-PDIA3 was transformed into *Escherichia coli* BL21 cells and confirmed. Proteins purified using the Ni^b+^  → Ni^2+^-nitrilotriacetic acid sepharose affinity column with PD-10 column chromatography (Amersham) were processed using Detoxi-Gel™ endotoxin removal gel (Pierce, Rockford, IL, USA).

### Tissue Processing and Immunostaining

The animals (n = 5 in each group) were perfused with 0.1 M phosphate‐buffered saline (PBS, pH 7.4), followed by 4% paraformaldehyde in 0.1 M phosphate buffer (PB, pH 7.4). The mouse brains were dissected and fixed in 4% paraformaldehyde for 12 h at 4 °C. Then, the brains were incubated overnight in 30% sucrose solution. Next, 30-μm-thick sections of the brains were obtained with a cryostat (Leica, Wetzlar, Germany) and stored in a preservative containing 30% glycerol, 30% ethyl glycol, and 10% 2PO_4_ in DW at 4 °C until use. The sections were subjected to overnight immunostaining at 4 ℃ with the following primary antibodies: rabbit anti‐Ki67 (1:1,000; Abcam, Cambridge, UK), rabbit anti-doublecortin (DCX, 1:2,000; Abcam), rabbit anti-myelin basic protein (MBP, 1:1,000; Abcam), rabbit anti-Iba-1 (1:500; Wako, Osaka, Japan), rabbit anti‐phosphorylated cAMP response element binding protein at Ser133 (pCREB, 1:1,000; Cell Signaling Technology Inc., Beverly, MA, USA), mouse anti-CRYM (1:250; Santa Cruz Biotechnology Inc., Santa Cruz, CA, USA), and rabbit anti-PDIA3 (1:1000; Thermo Fisher Scientific; Invitrogen, Waltham, MA, USA).

### Western Blotting

The animals (n = 5 in each group) were deeply anesthetized and the mouse brains were quickly removed from skull. The hippocampal tissues were obtained, homogenized, and lysed in RIPA buffer (ELPIS BIOTECH, Daejeon, Korea) according to the manufacturer's instructions. Equal amounts of proteins were loaded onto SDS-PAGE and transferred to a nitrocellulose membrane (Pall Life Sciences, Ann Arbor, MI, USA). The membrane was subjected to overnight immunostaining at 4 ℃ with the following primary antibodies: rabbit anti-DCX (1:2,000; Abcam), rabbit anti-MBP (1:1,000; Abcam), rabbit anti-Iba-1 (1:500; Wako), and rabbit anti‐pCREB (1:1,000; Cell Signaling Technology Inc.). Thereafter, the membrane was incubated with horseradish peroxidase-conjugated anti-rabbit IgG (1:10,000; Cell Signaling Technology Inc.) at 4 ℃ for 1 h and the protein bands were detected using chemiluminescence according to the manufacturer's instructions (Millipore, MA, USA).

### Data Quantification and Statistical Analysis

All immunoreactive structures were analyzed using ImageJ 1.53 (NIH, Bethesda, MD, USA). Four sections of the brain located between -1.70 and -2.46 mm from the bregma at 180 μm intervals were used to analyze the number or immunoreactivity of the immunohistochemically stained structures [26].

The whole hippocampus was regionally divided into CA1, CA2/3, and DG and optical densities of CRYM, PDIA3, MBP, Iba-1, and DCX were evaluated as the sum of gray scale (0–255) and pixel number using ImageJ. The number of Ki67- and pCREB-immunoreactive nuclei was in the SGZ of DG bounded by the GCL was determined. The relative optical density (ROD) was described as the percentage of the control value. The resultant data were represented as mean ± standard deviation and statistically analyzed using GraphPad Prism 5.01 (GraphPad Software Inc., La Jolla, CA, USA), as previously described [17]. One-way analysis of variance, followed by Tukey’s post hoc test, was performed, and a *p* < 0.05 was considered significant.

## Result

### The Effect of Demyelination on Protein Profiles in the *Hippocampus*

**Fig. 1:**
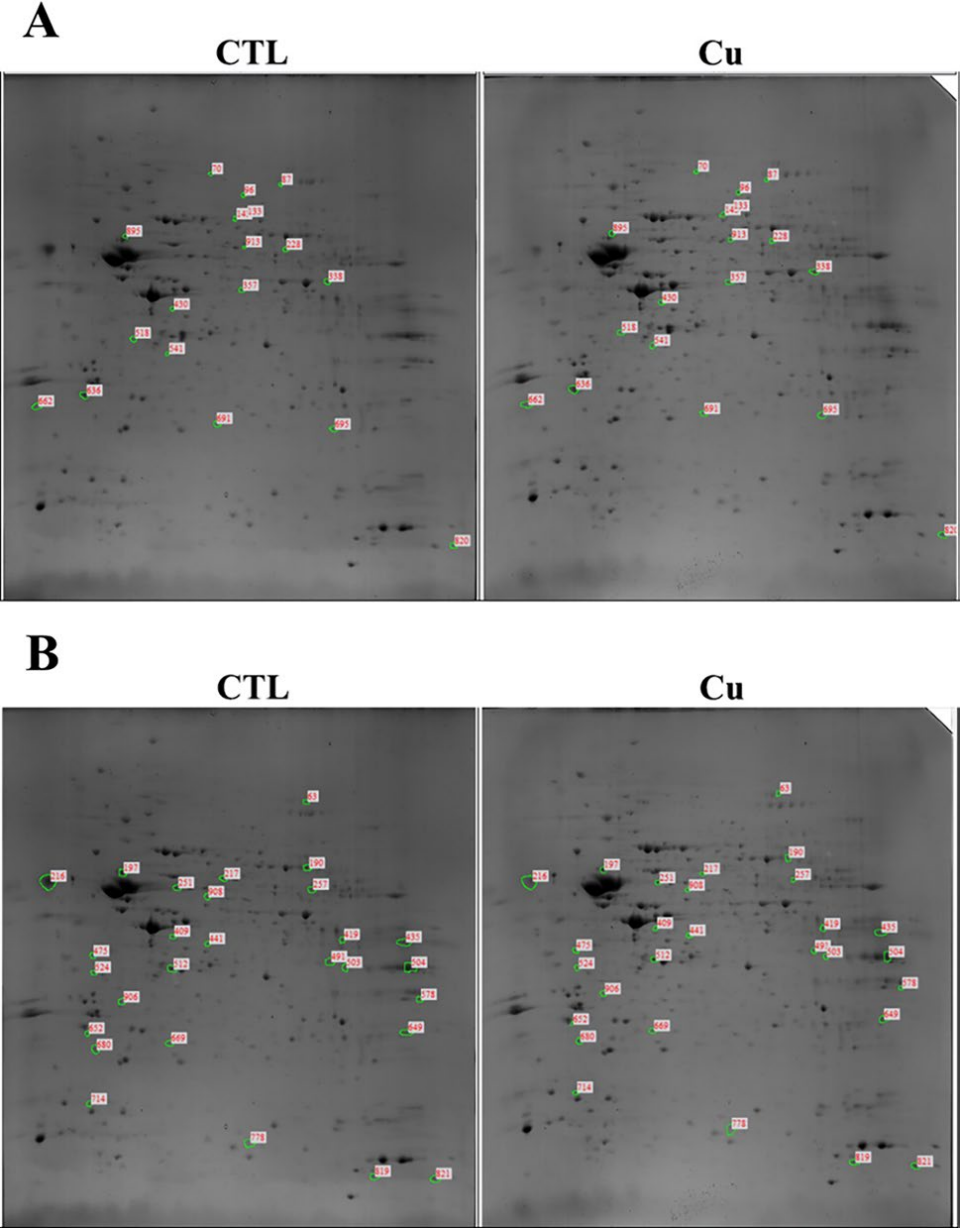
Spots on 2DE gels corresponding to hippocampal proteins extracted from group fed normal chow diet (CTL) and group fed CPZ diet (Cu) for 8 weeks. The density of spots in **A** was increased and that of spots in **B** was decreased by over two-folds in the Cu group compared with that in the CTL group

To explore the impact of demyelination on protein profiles in the hippocampus, we performed 2DE and analyzed protein changes following the administration and cessation of CPZ diet. In the CTL group, 556 spots were detected, while in the Cu group, 636 spots were detected on 2DE gels. Among these proteins, 509 spots were shared between the two groups, and respectively 18 and 28 spots in the Cu group showed more than two-fold increase and decrease in density compared with those in the CTL group (Fig. 1).

For protein identification, LC–MS/MS and MASCOT search methods were performed. Among the 18 and 28 spots, respectively 17 and 22 proteins were identified, details are presented in Tables 1A and B.

**Table 1:** List of identified proteins with increased or decreased expression in the group fed normal chow diet (CTL) or CPZ diet (Cu)

A. Increased							
Spot no	Accession no., protein name	Score	Mr (kDa)	pI value	Matched peptide	Sequence coverage (%)	Cu/CTL ratio
70	BAA08446.1, APG-1	69	95.285	5.53	2	1	2.06
87	BAC65593.1, mKIAA0567 protein, partial	100	64.566	8.87	2	3	2.48
96	NP_083949.2, MICOS complex subunit Mic60 isoform 1	1285	84.247	6.18	65	28	2.19
133	AAH06685.1, Trap1 protein, partial	46	80.370	6.25	1	1	2.09
143	NP_031534.2, V-type proton ATPase catalytic subunit A	120	55.958	5.64	3	4	2.17
228	EDL04922.1, Fyn proto-oncogene, isoform CRA_b	41	65.162	6.69	1	1	2.14
338	EDL27268.1, mCG127690	165	29.719	9.10	5	13	2.09
357	EDL31457.1, mitogen activated protein kinase kinase 2, isoform CRA_b	31	43,818	6.24	1	1	2.71
430	NP_032165.3, guanine nucleotide-binding protein G(q) subunit alpha	73	42.416	5.48	2	4	2.34
518	NP_080230.2, glycerol-3-phosphate phosphatase	134	34.975	5.24	4	8	3.07
541	NP_038499.2, annexin A4	86	36.178	5.43	2	6	2.48
636	ABL01512.1, beta-actin, partial	79	13.588	5.93	2	23	1.99
662	EDL02536.1, RAB5C, member RAS oncogene family, isoform CRA_b, partial	117	29.779	8.72	2	8	1.97
691	Q9DBP5.1, UMP-CMP kinase	37	22.379	5.68	1	6	2.32
820	EDL20118.1, mCG13600, isoform CRA_b	56	5.864	8.01	1	24	2.06
895	NP_058587.1, serine/threonine-protein phosphatase 2A 65 kDa regulatory subunit A alpha isoform	212	66.077	5.00	6	6	2.14
913	AAH18545.1, Hspd1 protein	34	27.112	4.76	1	3	2.11

B. Decreased							
Spot no	Accession no., protein name	Score	Mr (kDa)	pI value	Matched peptide	Sequence coverage (%)	CTL/Cu ratio
63	EDL24836.1, mCG6358	310	117.727	6.43	7	6	2.29
190	NP_034085.2, dihydropyrimidinase-related protein 2	138	62.638	5.95	3	5	4.42
197	EDL29822.1, guanosine diphosphate (GDP) dissociation inhibitor 1, isoform CRA_a, partial	1066	56.133	4.92	59	26	2.20
216	EDL04152.1, mCG18413, isoform CRA_b, partial	365	51.572	4.98	27	10	3.28
217	NP_031978.2, protein disulfide-isomerase A3 precursor	187	55.902	5.61	4	8	2.16
251	NP_476561.1, V-type proton ATPase subunit B, brain isoform	317	568.85	5.57	16	11	2.04
257	NP_038709.1, synapsin-2 isoform IIb	46	52.818	7.62	1	1	3.22
409	NP_001091.1, actin, alpha skeletal muscle	46	42.366	5.23	1	2	2.19
419	CAA30275.1, aspartate aminotransferase	342	46.489	6.68	8	16	2.11
435	NP_031769.2, collagen alpha-2(I) chain preproprotein	84	129.992	9.27	3	1	2.16
441	AAH11036.1, Bisphosphate 3'-nucleotidase 1	52	33.564	5.58	2	2	2.18
491	AGZ02590.1, sirtuin-2 isoform 3, partial	44	46.987	5.15	1	3	2.76
503	EDL00035.1, mCG117541	162	33.094	6.71	5	11	2.06
504	AAH92267.1, Glyceraldehyde-3-phosphate dehydrogenase	480	36.102	8.44	24	24	2.12
512	NP_057878.1, ketimine reductase mu-crystallin	791	33.673	5.44	38	31	2.13
524	NP_057878.1, ketimine reductase mu-crystallin	182	33.673	5.44	4	13	1.96
578	NP_031536.2, V-type proton ATPase subunit E 1	207	26.202	8.44	9	16	2.20
649	EDL01936.1, mCG131602, isoform CRA_a, partial	550	29.222	8.31	23	40	2.33
652	NP_003072.2, synaptosomal-associated protein 25 isoform SNAP25A	28	23.549	4.74	1	3	2.62
680	NP_004152.1 ras-related protein Rab-1A isoform 1	210	22.891	5.93	6	20	1.98
778	EDL23025.1 mCG5289	42	15.402	5.74	1	6	2.47
819	NP_032329.1 10 kDa heat shock protein, mitochondrial	76	10.956	7.93	2	23	2.43

### The Effect of Remyelination on Protein Profiles in the *Hippocampus*

**Fig. 2:**
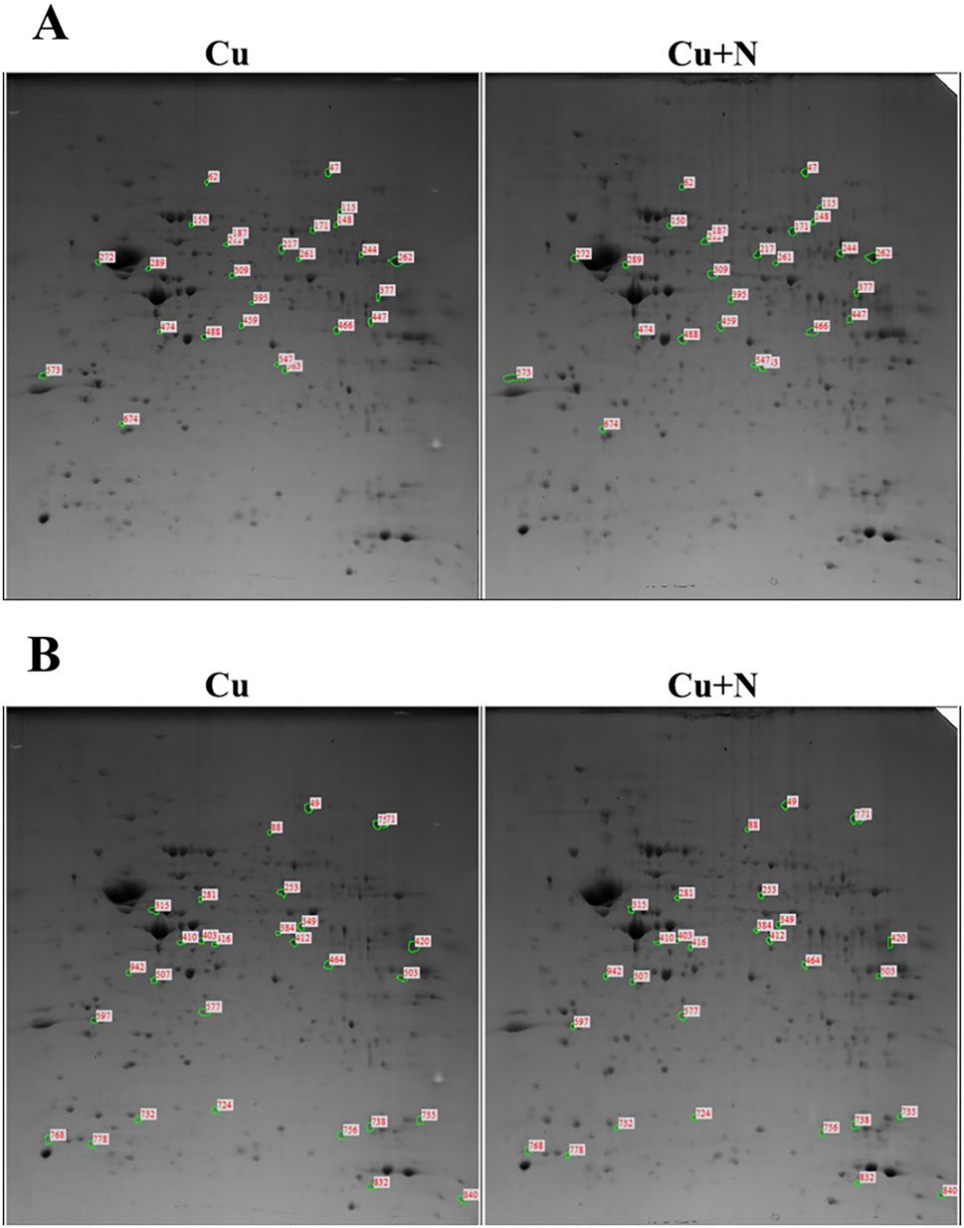
Spots on 2DE gel corresponding to hippocampal proteins extracted from the group fed CPZ diet (Cu) for 8 weeks and the group fed normal diet for 3 weeks after CPZ diet for 5 weeks (Cu + N). Spots in **A** showed increase and those in **B** showed decrease in density by over two-folds in the Cu + N group compared with that in the Cu group

To investigate the impact of remyelination on protein profiles in the hippocampus, 2DE was performed for the Cu and Cu + N groups. In the Cu and Cu + N groups, respectively 522 and 570 spots were detected; of these, 454 spots were shared between the two groups, and respectively 26 and 29 spots in the Cu + N group showed over two-fold increase and decrease in density compared with those in the Cu group (Fig. 2). Through LC–MS/MS and MASCOT search analysis, a total of 19 and 25 proteins were identified as presented as Tables 2A and B.

**Table 2:** List of identified proteins with increased or decreased expression in the group fed CPZ diet for 8 weeks (Cu) and the group fed normal diet for 3 weeks after CPZ diet for 5 weeks (Cu + N)

A. Increased							
Spot no	Accession no., protein name	Score	Mr (kDa)	pI value	Matched peptide	Sequence coverage (%)	Cu + N/Cu ratio
47	XP_006497712.2, PREDICTED: dynamin-1 isoform X1	2064	97.906	6.57	66	35	1.98
62	AAH94462.1, Aconitase 2, mitochondrial	323	86.036	8.08	8	8	2.22
150	EDL16325.1, p21 (CDKN1A)-activated kinase 1, isoform CRA_c	103	59.643	6.02	3	5	2.29
171	NP_034085.2, dihydropyrimidinase-related protein 2	279	62.638	5.95	16	10	2.77
187	NP_031663.1, T-complex protein 1 subunit epsilon isoform 1	223	60.023	5.69	4	6	2.24
212	NP_031978.2, protein disulfide-isomerase A3 precursor	765	57.099	5.88	24	30	2.44
217	NP_031662.2, T-complex protein 1 subunit beta	1001	57.783	5.97	25	25	3.19
244	NP_077150.1, succinyl-CoA:3-ketoacid coenzyme A transferase 1, mitochondrial precursor	75	52.636	8.98	2	4	2.21
262	NP_031531.1, ATP synthase subunit alpha, mitochondrial precursor	1972	59.83	9.22	103	43	2.07
272	NP_032950.1, cAMP-dependent protein kinase type II-alpha regulatory subunit	273	45.903	4.82	7	12	1.98
289	AAA37238.1, synexin	517	50.192	5.91	23	19	2.11
377	NP_032854.2, phosphoglycerate kinase 1	221	44.949	8.03	9	10	1.98
395	NP_033015.1, transcriptional activator protein Pur-alpha	84	34.976	6.07	3	3	2.74
447	EDL14930.1, acyl-CoA thioesterase 7, isoform CRA_b, partial	171	43.037	8.91	4	8	3.55
474	NP_057878.1, ketimine reductase mu-crystallin	296	33.673	5.44	7	18	2.68
488	EDL15010.1, guanine nucleotide binding protein, beta 1, isoform CRA_a, partial	140	38.494	5.61	3	7	2.26
547	EDK99781.1, myeloid leukemia factor 2, partial	108	25.922	7.29	5	10	2.14
573	NP_006752.1, 14–3-3 protein epsilon	459	29.326	4.63	15	36	2.21
674	AAH13897.1, Proteasome (prosome, macropain) subunit, beta type 6	198	21.995	5.14	4	14	2.24

B. Decreased							
Spot no	Accession no	Score	Mr (kDa)	pI value	Matched peptide	Sequence coverage (%)	Cu/Cu + N ratio
49	NP_034195.2, dynamin-1 isoform 1	394	97.603	7.23	10	8	3.28
71	AAH94462.1, Aconitase 2, mitochondrial	323	86.036	8.08	8	8	2.48
88	NP_033536.2, ezrin	116	69.506	5.9	3	3	1.99
253	NP_031662.2, T-complex protein 1 subunit beta	439	57.583	5.97	16	16	4.23
281	AQS27607.1, hypothetical protein	105	55.349	5.44	2	4	1.97
315	NP_034407.2, glial fibrillary acidic protein isoform 2	828	49.927	5.27	24	36	3.24
349	EDL06107.1, mCG21063, isoform CRA_a	213	44.923	6.09	6	10	2.65
384	AAH59848.1 Sept5 protein, partial	221	41.841	5.89	6	11	2.74
403	NP_036131.1, COP9 signalosome complex subunit 4 isoform a	112	46.541	5.57	2	5	1.98
410	NP_032165.3, guanine nucleotide-binding protein G(q) subunit alpha	427	42.416	5.48	10	23	2.66
412	NP_033015.1, transcriptional activator protein Pur-alpha	666	34.976	6.07	21	25	4.46
416	AAH11036.1, Bisphosphate 3'-nucleotidase 1	102	33.564	5.58	3	6	2.46
420	NP_031464.1, fructose-bisphosphate aldolase A isoform 2	768	39.787	8.31	25	28	2.62
464	NP_034818.1, LIM and SH3 domain protein 1	685	30.374	6.61	19	36	4.86
503	AAC26867.1, heterogenous nuclear ribonucleoprotein A2/B1	619	36.028	8.67	19	36	2.05
507	NP_062606.1, beta-soluble NSF attachment protein	196	33.878	5.32	6	12	2.04
577	AAI00416.1, Transcription elongation factor A (SII)-like 5	118	22.096	5.95	2	9	2.08
597	EDL08815.1, tyrosine 3-monooxygenase/tryptophan 5-monooxygenase activation protein, zeta polypeptide, isoform CRA_b, partial	806	29.24	4.71	22	38	2.61
724	BAB61894.1, stathmin	233	17.324	5.76	5	21	2.26
732	NP_001961.1, eukaryotic translation initiation factor 5A-1 isoform B	118	17.094	5.08	3	14	4.79
735	NP_031713.1, cofilin-1	178	18.776	8.22	4	19	5.00
756	EDL15910.1, mCG145251, partial	329	17.412	6.78	11	37	1.96
768	EDL02573.1, mCG144006, partial	137	55.01	5.14	4	5	2.05
832	NP_032329.1, 10 kDa heat shock protein, mitochondrial	89	10.956	7.93	2	21	3.40
942	NP_080230.2, glycerol-3-phosphate phosphatase	132	34.975	5.21	4	8	2.43

### Proteins with High Scores Identified in Both Proteomic Analyses

To identify reliable proteins among the candidates, the identified proteins in each group were sorted according to the following criteria: scores exceeding 70 and 20 in MASCOT search and sequence coverage, respectively. Proteins present in both lists of CTL *versus* Cu group and Cu *versus* Cu + N group are presented in Table 3. The expression of the 10 kDa mitochondrial heat shock protein was decreased in the CTL *versus* Cu group protein list as well as in the Cu *versus* Cu + N group protein list. Meanwhile, expression of the guanine nucleotide-binding protein G(q) subunit alpha was increased in the Cu group but decreased in the Cu + N group. Additionally, expression of PDIA3 and CRYM exhibited decreased expression levels in the Cu group and increased expression in the Cu + N group (Table 3).

**Table 3:** List of identical proteins in the protein lists

	Spot no	Accession no	Protein name	Score	Mr (kDa)	pI value	Matched peptide	Sequence coverage (%)	Ratio
CTL vs Cu	819	NP_032329.1	10 kDa heat shock protein, mitochondrial	76	10.956	7.93	2	23	−2.43
Cu vs Cu + N	832			89			2	21	−3.40

	Spot no	Accession no	Protein name	Score	Mr (kDa)	pI value	Matched peptide	Sequence coverage (%)	Ratio
CTL vs Cu	430	NP_032165.3	guanine nucleotide-binding protein G(q) subunit alpha	73	42.416	5.48	2	4	2.34
Cu vs Cu + N	410			427			10	23	−2.66

	Spot no	Accession no	Protein name	Score	Mr (kDa)	pI Value	Matched peptide	Sequence coverage (%)	Ratio
CTL vs Cu	217	NP_031978.2	protein disulfide-isomerase A3 precursor	187	57.099	5.88	4	8	−2.16
Cu vs Cu + N	212			765			24	30	2.44
CTL vs Cu	512	NP_057878.1	ketimine reductase mu-crystallin	791	33.673	5.44	38	31	−2.13
Cu vs Cu + N	474			296			7	18	2.68

### Selection and Validation of Proteins for Analysis

**Fig. 3:**
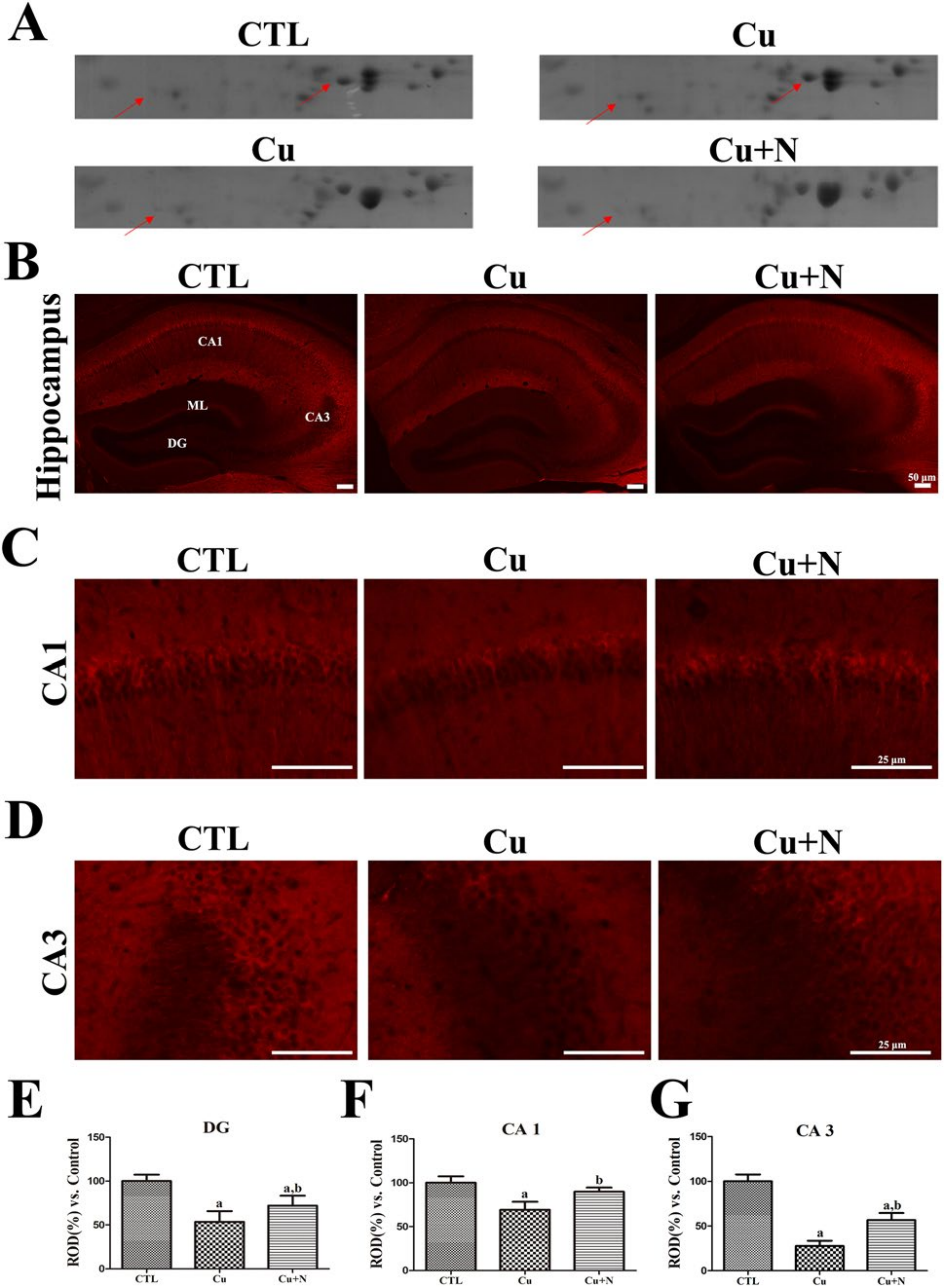
CRYM expression in the hippocampus of group fed normal chow diet for 8 weeks (CTL), group fed CPZ diet for 8 weeks (Cu), and group fed normal diet for 3 weeks after CPZ diet for 5 weeks (Cu + N). CRYM detected on **A** 2DE gels of CTL *versus* Cu group and Cu *versus* Cu + N group. CRYM immunoreactivity in **B** the whole hippocampus and magnified **C** CA1 and **D** CA3 regions in all groups. **E**, **F**, **G** ROD is described as the percentage of CRYM immunoreactivity in the CTL group. Scale bar = 50 μm **B** and 25 μm **C**, **D**. (n = 5 per group; ^a^p < 0.05, significantly different from the CTL group; ^b^p < 0.05, significantly different from the Cu group). All data are presented as mean ± standard deviation

After demyelination, the expression of CRYM and PDIA3 proteins decreased and then increased after remyelination. This suggests a close link to myelin survival or AHN. Notably, CRYM was detected at two different spots on 2DE gels in the CTL *versus* Cu group but at only one spot on 2DE gels in the Cu *versus* Cu + N group (Fig. 3A).

The expression of these proteins was validated through immunohistochemical staining. CRYM immunoreactivity was detected in the pyramidal layers of the hippocampal CA1 and CA3 regions and the ML of DG in the CTL group (Fig. 3B, 3C, and 3D). In the Cu group, weaker CRYM immunoreactivity was detected in the stratum pyramidale of CA1 and CA3 regions and ML of DG than that in the CTL group (Fig. 3B, 3C, and 3D). Specifically, CRYM immunoreactivity in DG, CA1, and CA3 in the Cu group was significantly decreased to 53.6%, 69.2%, and 27.7%, respectively, of that in the CTL group (Fig. 3E, 3F, and 3G). Moreover, CRYM immunoreactivity in DG, CA1, and CA3 in the Cu + N group was stronger than that in the Cu group (72.0%, 89.8%, and 56.6% of that in the CTL group) (Fig. 3E, 3F, and 3G). Meanwhile, CRYM immunoreactivity in DG, CA1, and CA3 in the Cu + N group was significantly stronger than that in the Cu group.

**Fig. 4:**
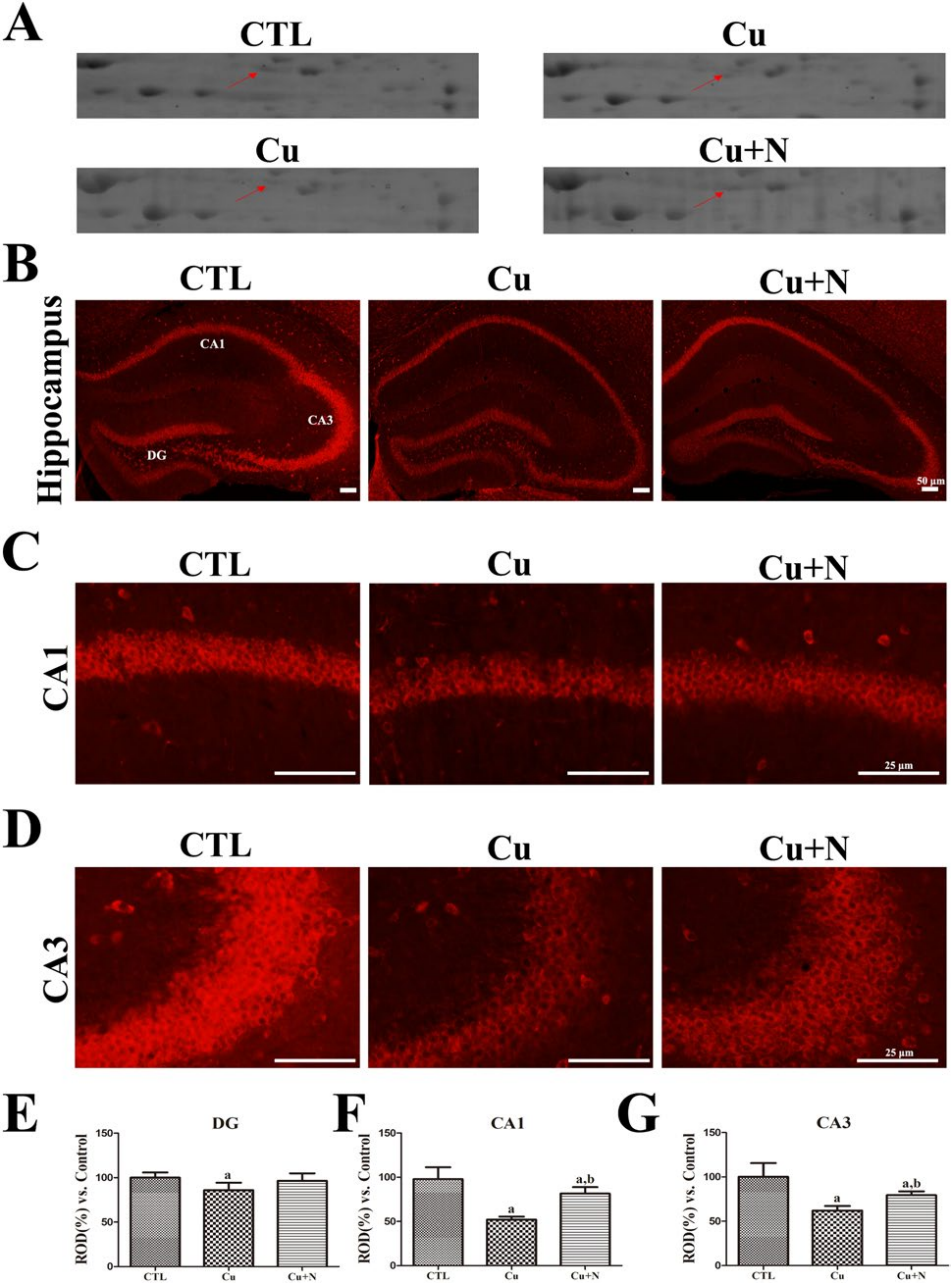
PDIA3 expression in the hippocampus in group fed normal chow diet for 8 weeks (CTL), group fed CPZ diet for 8 weeks (Cu), and group fed normal diet for 3 weeks after CPZ diet for 5 weeks (Cu + N). PDIA3 detected on **A** 2DE gels of CTL *versus* Cu group and Cu *versus* Cu + N group. PDIA3 immunohistochemistry in **B** the whole hippocampus and magnified **C** CA1 and **D** CA3 regions in all groups. **E**, **F**, **G** ROD is described as the percentage of PDIA3 immunoreactivity in the CTL group. Scale bar = 50 μm **B** and 25 μm **C**, **D**. (n = 5 per group; ^a^p < 0.05, significantly different from the CTL group; ^b^p < 0.05, significantly different from the Cu group). All data are presented as mean ± standard deviation

On 2DE gels, PDIA3 spots showed stronger density in the Cu + N group than in the Cu group (Fig. 4A). In the CTL group, PDIA3 immunoreactivity was primarily detected in the stratum pyramidale of CA1 and CA3 as well as in the GCL of DG (Fig. 4B, 4C, and 4D). In the Cu group, the distribution pattern of PDIA3 spots was similar to that in the CTL group, although immunoreactivity in DG, CA1, and CA3 in this group was significantly decreased compared with that in the CTL group (86.0%, 52.1% and 62.1%, respectively) (Fig. 4E, 4F, and 4G). In the Cu + N group, PDIA3 immunoreactivity in all regions was stronger than that in the Cu group (96.4%, 81.6%, and 79.4% of that in the CTL group, respectively) (Fig. 4E, 4F, and 4G). In the Cu + N group, PDIA3 immunoreactivity in CA1 and CA3 was significantly stronger than that in the Cu group.

### Effects of Tat-CRYM and Tat-PDIA3 on CPZ-Induced Demyelination in the Mouse *Hippocampus*

*CRYM* and *PDIA3* genes were integrated to a Tat peptide expression vector to synthesize Tat-CRYM and Tat-PDIA3 fusion proteins, respectively, and the two fusion proteins were overexpressed in *E. coli*. For protein purification, Ni^b+^  → Ni^2+^-nitrilotriacetic acid sepharose affinity column and PD-10 column chromatography were applied. As consistent with a previous study [24], the purified Tat-PDIA3 and Tat-CRYM proteins were confirmed by western blotting and clear single bands were detected at ~ 37 and ~ 62 kDa, respectively (Data not shown).

**Fig. 5:**
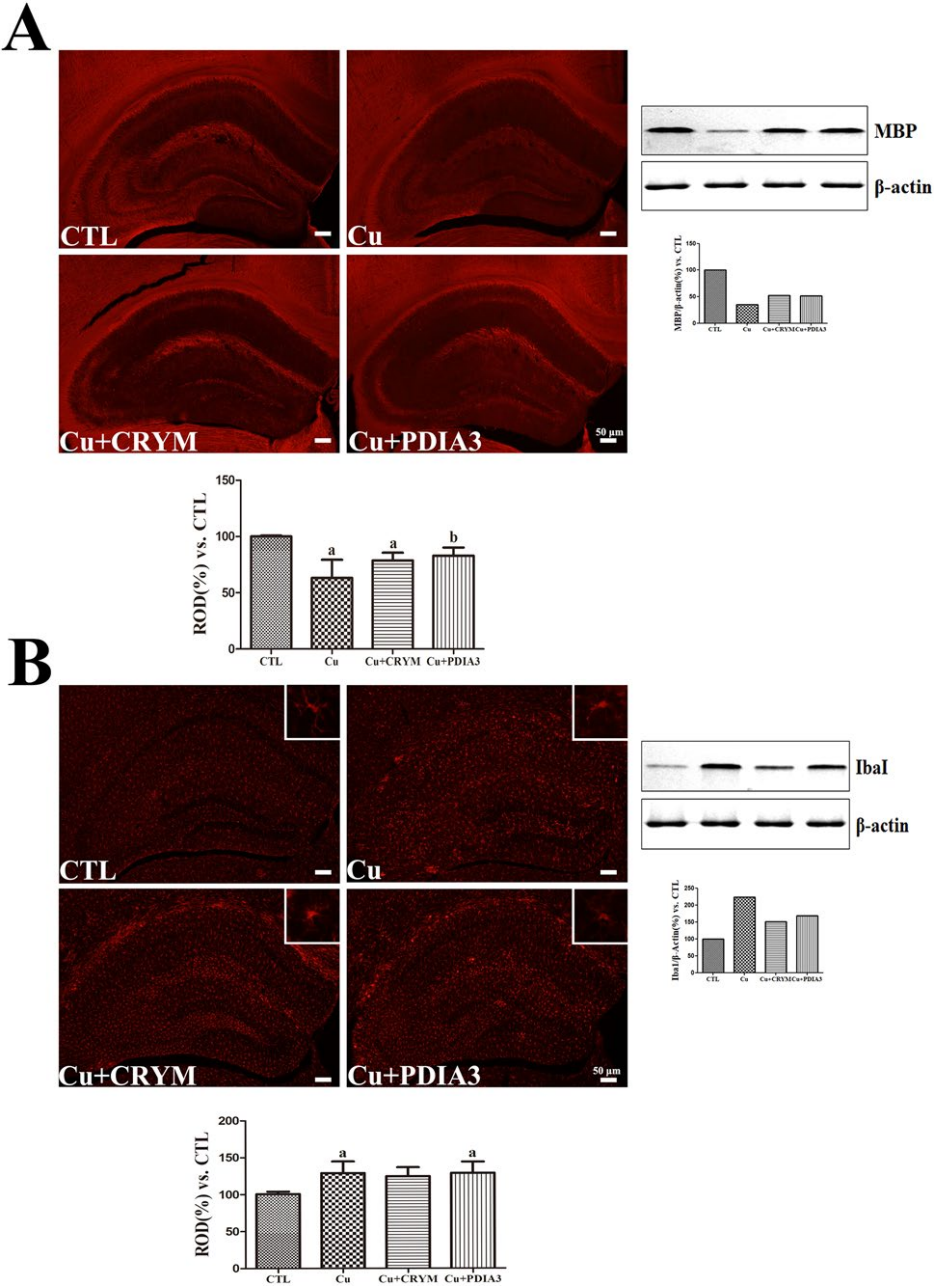
The expression and localization of MBP (**A**) and Iba-1 (**B**) were investigated using immunohistochemistry and Western blot analysis. In the hippocampus of group fed normal chow diet (CTL), group fed CPZ diet (Cu), group fed CPZ diet and administered Tat-CRYM injection (Cu + CRYM), and group fed CPZ diet and administered Tat-PDIA3 injection (Cu + PDIA3). ROD is described as the percentage of MBP or Iba-1 immunoreactivity in the hippocampus in the CTL group, respectively. Scale bar = 50 μm. (n = 5 per group; ^a^*p* < 0.05, significantly different from the CTL group; ^b^*p* < 0.05, significantly different from the Cu group). All data are presented as mean ± standard deviation

To evaluate the impact of Tat-CRYM and Tat-PDIA3 on demyelination, MBP immunohistochemical staining and western blot analyses were performed. In the CTL group, MBP immunoreactivity was detected in alveus and stratum-lacunosum moleculare of the CA1 region, mossy fibers of the CA3 region, and SGZ of the DG. In the Cu group, weak MBP immunoreactivity was detected in all hippocampal regions, and MBP immunoreactivity in the Cu group was significantly reduced (43.3% of CTL) compared with that in the CTL group. In the Cu + CRYM and Cu + PDIA3 groups, the distribution pattern of MBP immunoreactive structures was the same as that in the Cu group, although immunoreactivity was higher in these groups by 70.1% and 73.8% of that in the CTL group. However, the difference achieved statistical significance only between the Cu + PDIA3 and Cu groups (Fig. 5A). Protein expression levels were assessed using Western blot analysis. As illustrated in Fig. 5A, the expression of MBP was markedly reduced in the Cu group compared to the other groups. These results align with the immunohistochemistry findings, which also showed a significant decrease in MBP expression in the Cu group.

### Effects of Tat-CRYM and Tat-PDIA3 on CPZ-Induced Microglial Activation in the Mouse *Hippocampus*

Iba-1 immunostaining was performed to examine the effects of Tat-CRYM and Tat-PDIA3 on changes in microglial morphology in the hippocampus under CPZ-induced demyelination. In the CTL group, Iba-1-immunoreactive microglia were detected in all areas of the hippocampus, and their cytoplasm was small, with long processes. In the Cu group, Iba-1-immunoreactive microglia presented hypertrophied cytoplasm with thickened processes, and their immunoreactivity was significantly increased to 129.1% of that in the CTL group. In the Cu + CRYM and Cu + PDIA3 groups, the morphology of Iba-1-immunoreactive microglia was the same as that in the Cu group, but Iba-1 immunoreactivity was increased to respectively 124.8% and 129.1% of that in the CTL group; no significant differences were noted between the Cu + CRYM and Cu + PDIA3 groups and the Cu group (Fig. 5B). The Western blot analysis revealed an upregulation of Iba-1 in the treatment group compared to the control group. These results were consistent with the findings from the immunohistochemistry analysis, further confirming the increased expression of Iba-1 in the treatment group (Fig. 5B).

### Effects of Tat-CRYM and Tat-PDIA3 on CPZ-Induced Reduction in Cell Proliferation

**Fig. 6:**
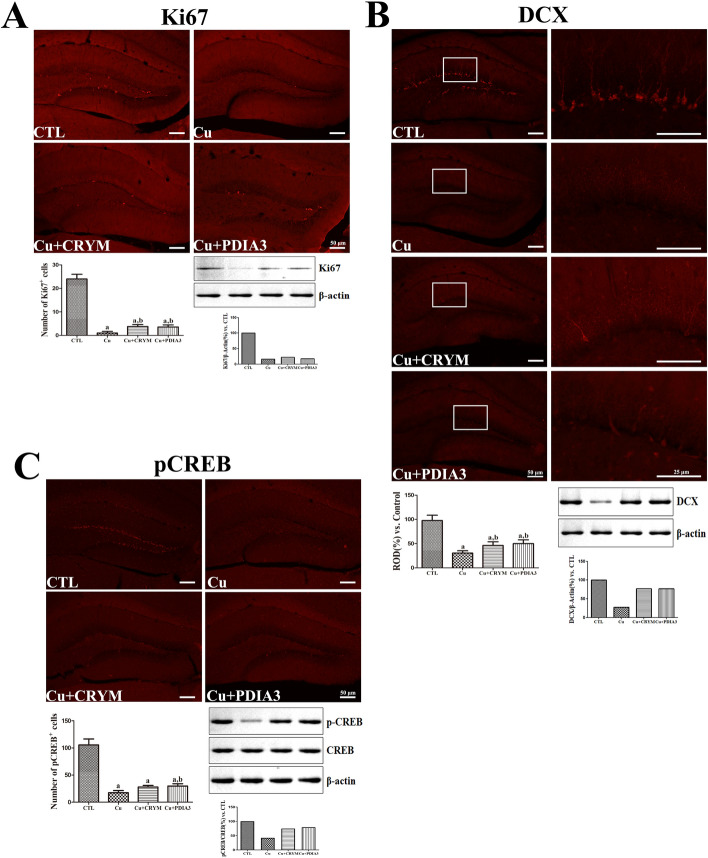
The expression and localization of **A** Ki67, **B** DCX and **C** pCREB were investigated using immunohistochemistry and Western blot analysis. In the hippocampal DG of group fed normal chow diet (CTL), group fed CPZ diet (Cu), group fed CPZ diet and administered Tat-CRYM injection (Cu + CRYM), and group fed CPZ diet and administered Tat-PDIA3 injection (Cu + PDIA3). The mean number of Ki67- and pCREB-positive nuclei in the SGZ of DG is shown. ROD is described as the percentage of DCX immunoreactivity in the hippocampus in the CTL group. Scale bar = 50 μm (n = 5 per group; ^a^*p* < 0.05, significantly different from the CTL group; ^b^*p* < 0.05, significantly different from the Cu group). All data are presented as mean ± standard deviation

Ki67 immunostaining was performed to observe proliferating cells in the DG. In the CTL group, Ki67-positive nuclei were detected in the SGZ of DG, and their mean number was 24 per section. In the Cu group, few Ki67-positive nuclei were detected in the SGZ, and their mean number was significantly reduced to 1 per section compared with that in the CTL group. In the Cu + CRYM and Cu + PDIA3 groups, a few Ki67-positive nuclei were detected in the SGZ of DG, and their number was significant increased (to 3.8 and 3.6 per section, respectively) compared with that in the Cu group. The Western blot analysis of Ki67 demonstrated that the other experimental groups exhibited lower levels of Ki67 protein expression compared to the control group. These findings suggest that the treatment induces a regulatory effect on Ki67 expression, which was similarly observed in the immunohistochemistry results (Fig. 6A).

### Effects of Tat-CRYM and Tat-PDIA3 on CPZ-Induced Reduction in Neuroblast Differentiation

In the CTL group, DCX-immunoreactive neuroblasts were detected in the SGZ of the DG, and their dendrites extended to the ML of the DG. In the Cu group, weaker DCX immunoreactivity was detected in the DG, and it was significantly decreased to 30.4% of that in the CTL group. In the Cu + CRYM and Cu + PDIA3 groups, a few DCX-immunoreactive neuroblasts were observed in the DG, and their dendrites were poorly developed than in those of neuroblasts in the CTL group. However, in these groups, DCX immunoreactivity was significantly increased compared with that in the Cu group (respectively 46.2% and 49.93% of that in the CTL group). The Western blot analysis for DCX revealed that the band intensity was reduced in the Cu group compared to the control group. Furthermore, the treatment groups exhibited an increase in band intensity relative to the Cu group, indicating an upregulation of DCX expression due to the treatment (Fig. 6B).

### Effects of Tat-CRYM and Tat-PDIA3 on CPZ-Induced Reduction in CREB Phosphorylation at Ser133 in the Moue *Hippocampus*

Phosphorylation of CREB was observed in the DG of hippocampus. Abundant pCREB-positive nuclei were detected in the CTL group, and their mean number was 105.6 per section. In the remaining groups, some pCREB-positive nuclei were detected in the SGZ of DG, and their mean number was 17.6, 28.0, and 29.8 per section, respectively, indicating significantly reduction in the number compared with that in the CTL group. Only in the Cu + PDIA3 group, the number of pCREB-positive nuclei was significantly higher than that in the Cu group (Fig. 6C). Western blot analysis was performed to evaluate the levels of pCREB and CREB in the DG of the hippocampus. The results were expressed as a percentage of the control group's immunoblot band values. While Western blot analysis showed similar levels of CREB protein across all groups, the pCREB/CREB protein levels varied (Fig. 6C).

## Discussion

MS is defined by an autoimmune-mediated attack on the myelin sheath, which disrupts the proper nerve signal transmission and leading to extensive demyelination in the hippocampus, among other areas [27]. This parallels our use of the CPZ model, which induces similar myelin damage and oligodendrocyte loss, closely resembling gray matter demyelinating diseases like MS [28]. This process results in significant demyelination, particularly within the hippocampus, a region crucial for learning and memory. The existing research indicates that promoting neurogenesis may offer therapeutic benefits for neurodegenerative conditions such as MS [29].

Due to its association with incomplete remyelination and remyelination failure, the primary focus of MS treatment is on achieving complete remyelination. Exposure of a CPZ diet for five week suppressed mitochondrial activity in the hippocampus while promoting the release of inflammatory cytokines like interleukin 1β and tumor necrosis factor-α [30]. Switching from the CPZ diet to normal chow after six weeks led to over 60% recovery of the myelin sheath in the hippocampus [31]. During remyelination, reports have shown myelin synthesis taking place in the hippocampus. Furthermore, neural progenitor cells play a role in maturing oligodendrocytes within the hippocampus [32, 33]. Various protocols and durations of demyelination and remyelination have been employed in studies on CPZ treatment.

Proteomic analysis is one of the useful approaches to identify therapeutic targets for diseases [34]. However, there have been no comprehensive studies aimed at detecting the possible targets for CPZ-induced demyelination and remyelination in the hippocampus. Therefore, in the present experiment, proteins whose expression patterns were altered by over two-folds after demyelination and remyelination were investigated through 2DE and LC–MS/MS.

CRYM was decreased in the CPZ group which is involved in NADPH binding [35] and thyroid hormone catabolism [36]. The expression of other protein, PDIA3, was increased in the group switched to normal diet after CPZ diet. PDIA3 activity is part of glycoprotein-specific quality control machinery involved in the folding of cysteine-rich glycoproteins and serves an important function in cellular signaling [37].

Furthermore, among these identified proteins, CRYM and PDIA3, were observed to have decreased expression during demyelination and increased expression during remyelination. This differential expression pattern implicates these proteins in the pathology of myelin sheath damage. Thus, targeting CRYM and PDIA3 may provide a viable strategy to alleviate the demyelination damage induced by CPZ.

CRYM is a protein belonging to the crystallin family and it is initially discovered in the eye lens. CRYM demonstrates widespread expression in various tissues, including the brain, kidney, and liver. Notably, in the brain, CRYM has been implicated in neuroprotection, acting as a safeguard for neurons against oxidative stress [38].

PDI is an enzyme involved in the formation, rearrangement, and cleavage of disulfide bonds during protein folding in the endoplasmic reticulum of cells and is upregulated in various neurological disorders, including Parkinson's disease and Alzheimer's disease [39]. PDIA3, also known as ERp57, is a redox chaperone and belongs to the PDI family of multifunctional proteins that play multiple roles in protein folding as well as calcium homeostasis, cell surface receptor regulation, and redox signaling pathways [40].

Accordingly, CRYM and PDIA3 in the hippocampus were analyzed as the candidates using immunostaining, and consistent results were obtained. During the process of demyelination, CRYM immunoreactivity was observed to decrease in the hippocampal DG, CA1, and CA3 regions. Conversely, during remyelination, an increase in CRYM immunoreactivity was noted in the same DG, CA1, and CA3 areas. According to previous studies, CRYM expression peaks at first and then decreases following extensive expression in immature neurons of hippocampal CA1 after birth [38]. In the present experiment, CRYM, which was upregulated in hippocampal CA1 after remyelination, may play a similar role during hippocampal neuronal development. Furthermore, PDIA3 immunoreactivity was decreased in DG, CA1, and CA3 in the demyelinated state and increased in CA1 and CA3 in the remyelinated state. The increase in PDIA3 expression in the remyelinated state in the present experiment is supported by a previous finding that mutant PDIA3 exhibited impaired neurogenesis function [41]. PDIA3 and CRYM immunoreactivity was significantly increased in the hippocampal CA1 region during remyelination. Therefore, immunohistochemical results for PDIA3 and CRYM in the hippocampus indicated that the expression of these proteins decreases upon demyelination and increases upon remyelination. Overall, the results of proteomic analysis and protein validation were consistent. We confirmed that the expression of CRYM and PDIA3 decreased during demyelination and increased during remyelination. However, the effects of Tat-CRYM or Tat-PDIA3 on myelin damage, cell proliferation, neuroblast differentiation, and CREB phosphorylation in the hippocampus during demyelination or remyelination remain unknown.

As long-term (8 weeks) exposure to CPZ induces strong demyelination in the brain [28], CPZ was administered for 8 weeks. In addition, neuroblasts and immature neurons have been reported to express DCX by 4 weeks after birth [42].

The elucidate the effect of Tat-CRYM or Tat-PDIA3 on CPZ-induced demyelination, immunohistochemical staining of MBP, the major component of myelin sheath, was performed. In the present study, only Tat-PDIA3 efficiently ameliorated CPZ-induced demyelination in the hippocampus.

Next, inflammatory response was visualized to detect microglia in the hippocampus, because microglia, which are involved in the inflammatory response of the central nervous system, are one of the major cell types [43]. These cells contribute to brain damage repair and brain development under normal physiological conditions [44, 45]. CPZ-induced demyelination activates microglia, with hypertrophied cytoplasm and thickened processes. Likewise, abnormal microglial activation has been observed in several neurodegenerative diseases that affect cognition and memory, such as Parkinson’s and Alzheimer’s diseases [46, 47]. However, in the present study, no CPZ-induced microglial activation was observed following Tat-PDIA3 and Tat-CRYM treatment.

To assess the effects of Tat-PDIA3 and Tat-CRYM on CPZ-induced reduction in AHN, proliferating cells and differentiated neuroblasts were visualized through immunohistochemical staining for Ki67 and DCX. Consistent with previous reports [13, 17], CPZ diet reduced the number of proliferating cells and differentiated neuroblasts in the DG. This result is consistent with previous studies that cuprizone-induced demyelination causes the shift the cell state from proliferation to quiescence in radial glia-like type 1 neural stem cells [48] and the reduction of differentiated neuroblasts in the DG [17, 21, 33, 49]. However, Tat-PDIA3 and Tat-CRYM treatment significantly mitigated these effects in the DG even during demyelination. These findings are supported by a previous report that Tat-PDIA3 administration further increased the number of proliferating cells and neuroblast in the hippocampal DG under ischemic damage [25].

The maturation, survival, and integration of newly generated neurons in the hippocampus is controlled by CREB-dependent signaling [50], and the number of proliferating cells and neuroblasts is positively correlated with pCREB levels in the hippocampus after CPZ administration [13, 17]. CPZ-induced demyelination significantly decreased the number of pCREB-positive nuclei in the SGZ of DG, while only Tat-PDIA3 treatment exerted an ameliorative effect on the suppression of CREB signaling under demyelination.

In previous studies, downregulation of CRYM mRNA was reported in animal models of neurodegenerative disorders [51, 52], and CRYM was reported to exert neuroprotective effects in the striatum in a mouse model of Huntington’s disease [53]. In addition, PDIA3 downregulation led to acute motor dysfunction with synaptic loss [54]. Furthermore, neuroprotective effects of PDIA3 against toxicity induced by methamphetamine, a neurotoxic drug, have been demonstrated in a neuroblastoma cell line [55]. Thus, increased CRYM and PDIA3 expression may be the one of the compensatory mechanisms to protect the neurons from demyelination induced by CPZ exposure. Taken together, the improvement of neurogenesis and CREB signaling suggests that PDIA3 serves as an essential modulation target in hippocampal neurogenesis during demyelination and remyelination.

In the present study, the mechanism of Tat-PDIA1 remains to be elucidated, although several hypotheses can be proposed. As the PDI family is involved in redox folding, the misfolded substrates go through the refolding cycle in conjunction with another chaperone calnexin (CNX) [56]. In particular, PDIA3 is involved in the oxidative folding of CNX and glycoproteins [57], and deficiency of this protein may lead to the misfolding and dysfunction of myelin protein zero, which is one of the major myelin components [58]. Additionally, a previous study has shown that CNX is involved in the folding of myelin proteins [59]. Therefore, PDIA3 and CNX are synergistically involved in myelin regeneration and, ultimately, nerve regeneration. Corroborating the speculations of Castillo et al. (2015) that PDIA3 is a central component of the CNX–calreticulin cycle and serves relevant functions in maintaining glial protein degradation, this protein may contribute to the maintenance of the myelin structure [60].

CPZ induced demyelination and activated microglia in the hippocampus, significantly suppressing cell proliferation, neuroblast differentiation, and CREB phosphorylation in the DG. Meanwhile, switch to normal diet from CPZ diet reversed the damage to the myelin sheath and suppression of cell proliferation, neuroblast differentiation, and CREB phosphorylation.

Further, significantly altered protein profiles were assessed through 2DE and LC–MS/MS after the intake of CPZ diet and after switch to normal diet. Specifically, the expression of CRYM and PDIA3 was decreased after the intake of CPZ diet and increased after switch to normal diet. Consistent with the findings of proteomic analyses, the expression of PDIA3 and CRYM in the hippocampal DG was significantly decreased during demyelination and increased during remyelination.

After validating the above results, the Tat-CRYM and Tat-PDIA3 proteins were prepared. Treatment with both proteins alleviated the suppression of cell proliferation and neuroblast differentiation in the hippocampus, while treatment with Tat-PDIA3 alone mitigated the damage to the myelin sheath and suppression of CREB signaling during demyelination.

These findings suggest that chaperone proteins like PDIA3 may have therapeutic potentials in mitigating the impairments in adult neurogenesis induced by demyelination. Considering the crucial role of chaperones in protein folding and misfolding processes, they may represent a promising avenue for addressing the neurodegenerative aspects of MS. Furthermore, the hippocampus, given its involvement in cognitive functions, is a significant area of study in the context of MS and demyelination-induced damage in the hippocampus can contribute to the cognitive deficits commonly observed in MS patients. The changes of adult neurogenesis induced by CPZ in the hippocampus and amelioration by PDIA3 provides valuable insights into how the processes of demyelination and remyelination in this region might be implicated in the pathology and potential therapeutic interventions for MS.

## Conclusion

Our study demonstrates that CPZ exposure induces demyelination and microglial activation in the hippocampus, leading to significant suppression of cell proliferation, neuroblast differentiation, and CREB phosphorylation in the DG. Switching to a normal diet from a CPZ diet reversed these effects, promoting remyelination and recovery of cellular functions. Proteomic analyses confirmed that proteins CRYM and PDIA3 are significantly modulated during these processes. Moreover, treatment with Tat-CRYM and Tat-PDIA3 proteins alleviated the negative effects of CPZ, with Tat-PDIA3 showing particular promise in mitigating demyelination-induced impairments. These findings highlight potential therapeutic targets for treating demyelination-related neurological conditions.

## Data Availability

No datasets were generated or analysed during the current study.
